# Predictive Modeling of Channel Catfish Under Varying Temperatures: Quality Dynamics and Warning Thresholds

**DOI:** 10.3390/foods15091557

**Published:** 2026-04-30

**Authors:** Hongyu Jiang, Wang Li, Binchen Wang, Enhao Yao, Yingxi Chen, Sufang Zhang, Beiwei Zhu

**Affiliations:** SKL of Marine Food Processing & Safety Control, National Engineering Research Center of Seafood, Collaborative Innovation Center of Seafood Deep Processing, School of Food Science and Technology, Dalian Polytechnic University, Dalian 116034, China

**Keywords:** channel catfish, total viable count, volatile basic nitrogen, kinetic model, artificial neural network, quality dynamics

## Abstract

The objective of this work was to establish mathematical models and an artificial neural network to predict changes in channel catfish quality during storage. Secondary models of microorganisms, using the total viable count (TVC) as an indicator, were established based on the modified Gompertz equation combined with the Belehradek equation. The secondary kinetic models for total volatile basic nitrogen (TVB-N) were developed by combining the primary model with the Arrhenius equation, from which the early warning thresholds for quality change were determined based on the slopes of the kinetic curves. For most samples, the relative error between the measured and predicted values of the secondary kinetic model remained within ±20% across the tested storage temperatures, while during the practically relevant 2–6 days period, the error was tightly controlled within ±15% for the majority of samples. Moreover, the prediction models were established based on Back Propagation Neural Networks and Radial Basis Function Neural Networks, with determination coefficients (R2) exceeding 0.9. In conclusion, the developed predictive models provide a scientific basis and technical support for quality monitoring and cold-chain distribution of channel catfish under varying temperatures.

## 1. Introduction

Channel catfish (Ictalurus punctatus) is regarded as a commercially significant freshwater species due to its tender flesh and nutritional value, and it is favored by consumers both domestically and internationally [1]. Nevertheless, channel catfish throughout the stages of transportation, storage, and sales were highly vulnerable to temperature fluctuations, which led to varying degrees of spoilage. Therefore, developing effective methods for monitoring and predicting quality changes is a critical task during the cold-chain distribution of channel catfish [2]. The total viable count (TVC) is a fundamental parameter for assessing the microbiological quality of aquatic products, as it reflects the growth and proliferation of microorganisms during storage. The increase in microbial populations is the primary cause of fish spoilage, as microorganisms actively metabolize proteins, lipids, and other nutrients, leading to quality deterioration [3]. Moreover, rapid microbial growth can significantly shorten the shelf life of aquatic products. Therefore, monitoring and predicting the growth dynamics of TVC are essential for evaluating product stability and ensuring food safety. The total volatile basic nitrogen (TVB-N), in contrast, reflects the accumulation of volatile nitrogenous compounds, such as ammonia, produced mainly through microbial activity and protein degradation [4]. It is widely regarded as a direct indicator of freshness in aquatic products, with its levels typically increasing in parallel with storage time and quality deterioration [5]. Therefore, the monitoring and prediction of TVB-N levels are crucial for determining shelf life and developing early warning systems for quality changes. Considering the close interrelationship between TVC and TVB-N, integrating these two indicators provides a more comprehensive understanding of spoilage processes and offers a reliable basis for shelf-life prediction and quality control in aquatic products [6].

Mathematical models are composed of formulas for obtaining output sample data from extensive input sample data, including microbial prediction models and chemical reaction kinetics prediction models. Kinetic models have proven effective for predicting quality changes in fish during storage and transportation and for establishing reliable early warning thresholds. Unlike traditional limit-based indicators that signal spoilage only after it occurs, early warning thresholds enable proactive intervention. These thresholds facilitate precise shelf-life management and informed decision-making in cold-chain logistics under fluctuating temperatures, positioning them as a key component of future predictive models for aquatic products.

The artificial neural network (ANN) serves as a mathematical algorithmic framework for information processing, emulating the functional attributes of the human nervous system [7]. Recently, there have been reports on both mathematical and neural network-based models aimed at predicting the quality of aquatic products. Zhang et al. [8] developed a predictive model for assessing the quality of grass carp at different storage temperatures by employing the Arrhenius equation. Liu et al. [9] performed a comparative study of the Arrhenius model and an ANN to predict changes in the quality of rainbow trout filets. Xu et al. [10] conducted a comparative analysis between the Arrhenius model and a radial basis function neural network (RBFNN) model for predicting quality changes in frozen prawns. Although both ANNs and mathematical models have been widely employed in aquatic product quality prediction, studies specifically addressing quality variation across different anatomical regions of channel catfish remain scarce. Furthermore, aquatic products often experience frequent temperature fluctuations in real-world distribution environments. However, the majority of the previous models developed were derived under stable thermal conditions [11,12,13]. Therefore, it is practically important to establish microbial kinetic models that account for dynamic temperature conditions. However, in real-world production and distribution environments, aquatic products are often subjected to significant and frequent temperature fluctuations. Therefore, establishing microbial kinetic models that account for dynamic temperature conditions is of great practical importance. The modified Gompertz model has been widely applied due to its strong capability in describing microbial growth characteristics, particularly the lag phase and maximum growth rate. On this basis, the Belehradek equation can be introduced as a secondary model to characterize the effect of temperature on kinetic parameters. This combined approach improves model applicability under varying storage temperatures while maintaining relative simplicity [14].

In this study, we assessed the quality changes in different anatomical segments of channel catfish during storage at temperatures ranging from 0 to 15 °C. Predictive and early warning models for total viable count (TVC) and total volatile basic nitrogen (TVB-N) were developed using the modified Gompertz model, the Arrhenius equation, a backpropagation neural network (BPNN), and a radial basis function neural network (RBFNN). Meanwhile, a comprehensive comparative evaluation is necessary to identify the superior model for different anatomical segments of channel catfish. This work aims to support real-time quality monitoring of segmented channel catfish products during storage and transportation, providing a practical reference for ensuring product quality and safety.

## 2. Materials and Methods

### 2.1. Raw Materials and Pretreatment

The experiment was conducted in accordance with the guidelines established by the China State Council in 1988 and the Guidance on Treating Experimental Animals of the country’s Ministry of Science and Technology in 2006. Channel catfish (average weight 2.0 kg) were procured from a local aquatic market in Dalian, China, and dispatched to the laboratory in oxygenated water. Upon reception, channel catfish samples were promptly euthanized using percussive stunning. Subsequently, the channel catfish were processed with open backs, gutted, and thoroughly cleaned. Based on the anatomical position of the fins, the fish were divided into five distinct sections: head muscle (HM), brisket muscle (BRM), belly muscle (BEM), dorsal muscle (DM), and tail muscle (TM) (Figure S1). Various segments of channel catfish were cut into approximately 1.0 cm^3^ fragments, enclosed in sterile polyethylene pouches, and stored under controlled conditions at 0, 4, 5, 10, and 15 °C with a constant relative humidity of 60% in a temperature–humidity incubator. During storage, samples were collected at predetermined intervals for analysis, specifically every 2 days under 0, 4, and 5 °C conditions, and every 12 h under 10 and 15 °C conditions. Prior to analysis, samples were removed from the pouches and equilibrated to room temperature. Additionally, a series of fluctuating temperature conditions was implemented: 4 °C 2 days → 9 °C 2 days (sampling point A) → 14 °C 1 day → 9 °C 2 days → 4 °C 2 days (sampling point B). In the experiment procedure, channel catfish samples were extracted at each time point for index analysis. Other reagents were sourced from Sinopharm Chemical Reagent Co., Ltd. (Shanghai, China). All reagents were of analytical grade purity.

### 2.2. Total Viable Count

The sample (1.0 g) was aseptically transferred into 9 mL of sterile saline (0.85% NaCl) and thoroughly homogenized to obtain an initial 10^−1^ dilution. Serial decimal dilutions were then prepared using sterile saline. From appropriate dilutions, 100 μL aliquots were aseptically spread onto plate count agar (PCA) plates using the spread plate method to ensure that plates with 30–300 colonies were obtained for enumeration. Each dilution was plated in triplicate. The plates were incubated at 30 °C for 72 h [15]. Plates containing 30–300 colonies were considered countable, and the average colony counts from triplicate plates were used to calculate microbial loads. The results were expressed as log (CFU/g) of the sample.

### 2.3. Total Volatile Basic Nitrogen

A sample (1.0 g) was combined with 10.0 mL of deionized water to create a homogenate, macerated for 30 min, and then centrifuged (4000 r/min, 5 min) to leave the supernatant aside. The water-soluble gel was applied to the edge of the diffusion dish. Subsequently, 1 mL of H_3_BO_3_ (20 g/L) and 1 drop of mixed indicator solution (comprising a 1 g/L methyl red ethanol solution mixed 1:1 with 5 g/L bromocresol green ethanol solution) were added to the inner chamber in the center of the dish. Then, 1.0 mL of the supernatant was transferred to the outer chamber of the dish. After sealing the dish with a frosted glass lid, 1.0 mL of saturated potassium carbonate solution was introduced. The setup was maintained at 37 °C for 2 h before being removed. Subsequently, the solution was titrated with 0.01 mol/L hydrochloric acid standard titrant [16]. All experiments were conducted in triplicate. The value of TVB-N was calculated as follows:

$$TVB-N(mg/100g)=\frac{\left(V1-V2\right)\;\times \;c\;\times \;14}{m\;\times \;10/25}\times 100$$

where *V*1 is the consumed volume of HCL standard solution for the titration; *V*2 is that of the blank control; *c* is the concentration of HCl; and *m* is the mass of each sample.

### 2.4. Modified Gompertz Equation Combined with Belehradek Modeling

The relationship between TVC and storage duration at different temperatures was described by the modified Gompertz model [17], a widely used sigmoid-type primary growth model for microbial proliferation. The model equation is shown in Equation (1) [18].

(1) $$N\left(t\right)=N_{0}+(N_{\max}-N_{0})\;\times \;\exp\{-\exp\left[\frac{\mu_{\max}\;\times \;2.718}{N_{\max}\;-\;N_{0}}\;\times \;\left(\lambda -t\right)+1\right]\}$$

where *t* is time (d), N(*t*) is the number of colonies (log (CFU/g)) at time *t*, N_0_ is the initial number of colonies (log (CFU/g)), N_max_ is the maximum number of colonies (log (CFU/g)), μ_max_ is the maximum specific growth rate (d^−1^), and λ is the lag phase duration (d).

The Belehradek equation (Square root model) [19] was employed as a secondary model to illustrate the correlation between temperature and kinetic parameter as shown in Equations (2) and (3):

(2) $$\sqrt{\mu_{\max}}\;={\;b}_{\mu }(T\;-\;T_{\min})$$

(3) $$\sqrt{\frac{1}{\lambda }}=b_{\lambda }(T\;-\;T_{\min})$$

where T stands for the storage temperature (°C), T_min_ is a theoretical parameter that represents the temperature at which microbial activity ceases. T_min_ was obtained by extending the regression line until it intersects the temperature axis, at which point the μ_max_ was zero.

Due to temperature fluctuations during the storage and transportation of aquatic products, the overall process can be subdivided into multiple isothermal processes [20].

When *t* = d*t*_1_,

(4) $$N\left(t_{1}\right)={\;N}_{0}+(N_{\max}\;-\;N_{0})\;\times \;\exp\{-\exp\left[\frac{\mu_{\max}\;\times \;2.718}{N_{\max}\;-\;N_{0}}\;\times \;\left(\lambda_{1}\;-\;t_{1}\right)+1\right]\}$$

When *t* = d*t*_1_ + d*t*_2_,

(5) $$N\left(t_{2}\right)=N_{1}+(N_{\max}\;-\;N_{1})\;\times \;\exp\{-\exp\left[\frac{\mu_{2}\;\times \;2.718}{N_{\max}\;-\;N_{1}}\;\times \;\left(\lambda_{2}\;-\;t_{2}\right)+1\right]\}$$

When *t* = d*t*_1_ + d*t*_2_ + … + d*t*_i_,

(6) $$N\left(t_{i}\right)=N_{i-1}+(N_{\max}\;-\;N_{i-1})\;\times \;\exp\{-\exp\left[\frac{\mu_{i}\;\times \;2.718}{N_{\max}\;-\;N_{i-1}}\;\times \;\left(\lambda_{i}\;-\;t_{i}\right)+1\right]\}$$

where d*t_i_*(i = 1,2,3…) denotes the hypothetical short time interval of constant temperature (d); *N*(*t_i_*) represents the microbial count at time d*t_i_* [log (CFU/g)]; *N*_0_ is the initial microbial population at t = 0 [log (CFU/g)]; and *N_max_* is the maximum microbial population attained in the stationary phase [log (CFU/g)]. In the present stepwise dynamic formulation, the lag time $\lambda_{i}$ was re-initialized for each temperature interval. This simplification treats every temperature shift as an independent thermal perturbation. This approach maintains model parsimony, and it does not account for any carry-over effect of the microbial adaptation state from the previous temperature segment.

### 2.5. Primary Chemical Reaction Kinetics Combined with Arrhenius Modeling

Alterations in fish quality throughout production and processing can be characterized by kinetic models. Most of the quality changes associated with fish products follow zero- or first-order reaction kinetics, with the first-order reaction kinetic model being extensively employed [21,22]. The equation is given by (7):

(7) $$B(t)\;=\;B_{0}e^{\mathrm{Kt}}$$

where B(*t*) is the TVB-N content at time *t* (mg/100 g), B_0_ is the initial TVB-N content (mg/100 g), K is the rate constant of TVB-N (constant), and *t* is the storage time (d).

To establish a quality early warning model for aquatic products, Equation (7) was differentiated to obtain the following expression (8). The early warning threshold was defined based on the slope value at which the curve exhibited a rapid increase. Considering the variation in kinetic behavior under different storage temperatures, temperature-specific slope thresholds were applied. The threshold slopes were set at 0.6 for 0 °C, 1.0 for 5 °C, 3.0 for 10 °C, and 5.0 for 15 °C, to ensure that the warning is triggered at comparable spoilage stages rather than at identical absolute rates.

(8) $$B^{\prime }(t)=B_{0}Ke^{Kt}$$

The Arrhenius equation for the reaction was derived at various storage temperatures to ascertain the number of reaction phases and the corresponding reaction constants [23]. The equation is given by (9)

(9) $$K=k_{0}\;\times \;\exp(-\frac{E_{a}}{\mathrm{RT}})$$

where k_0_ is the frequency factor, T is the absolute temperature (K), R is the gas constant, 8.3144 J/(mol·K), E_a_ is the activation energy (J/mol).

By employing Equations (7) and (9), the predictive model for shelf life (1.10) can be obtained [24]:

(10) $${\mathrm{SL}}_{\mathrm{TVB}-N}\;=\;\frac{\ln(\frac{B}{B_{0}})}{k_{0}\;\times \;\exp(-\frac{E_{a}}{\mathrm{RT}})}$$

where B is the TVB-N international standard value (20 mg/100 g).

Fluctuating temperatures during storage and transport can be approximated by a series of isothermal steps.

When *t* = d*t*_1_ + d*t*_2_ + …+ d*t*_i_,

(11) $$B(t_{i})=B_{i-1}e^{K_{i}t_{i}}$$

### 2.6. Establishment and Evaluation of the ANN Model

The predictive models of BPNN and RBFNN were developed utilizing the newff and newrbe functions in the MATLAB environment. All experimental data collected at temperatures of 0, 5, 10, and 15 °C were used for model training. Normalizing the data eliminated the effect of magnitude and sped up model calculations. A total of 300 samples (excluding the data at 4 °C condition) were generated from the TVC and TVB-N data of five channel catfish segments at different sampling times, with storage temperature and time as input variables and TVC/TVB-N values as output variables. The samples were randomly split at a 7:3 ratio, yielding 210 training samples and 90 independent testing samples. To ensure reproducibility, a fixed random seed (set to 48) was used for both data partitioning and network initialization. The BPNN model adopted a three-layer structure with 10 hidden neurons, using the tansig transfer function in the hidden layer and the purelin function in the output layer, and was trained using the trainlm algorithm. The stopping criteria were set to a maximum of 1000 epochs and a learning rate of 0.01. To reduce the influence of random initialization, each model was run 10 times, and the optimal model was selected based on the highest R^2^ and lowest RMSE. The RBFNN model was constructed using the newrbe function, which employs radial basis functions as activation units in the hidden layer. The spread parameter was optimized empirically to achieve a balance between fitting accuracy and generalization performance. Similar to the BPNN model, the RBFNN was trained using the same training dataset and evaluated on the independent testing set [25].

(12) $$X^{\prime }=\frac{X\;-\;X_{\min}}{X_{\max}\;-\;X_{\min}}$$

where X denotes the raw data, X′ denotes the normalized data, and X_max_ and X_min_ denote the maximum and minimum values of each variable.

The following performance metrics were employed for evaluation [26]: determination coefficients (R2), Relative Error (RE), Mean Bias Error (MBE), Mean Absolute Percentage Error (MAPE), and Root Mean Square Error (RMSE). The predictive performance was further assessed using experimental data by comparing the relative errors between the ANN-predicted values and the corresponding experimental values.

(13) R2=1 − ∑i=1N(yi − y^i)2∑i=1N(yi − y¯i)2

(14) RE(%)=yi − y^iyi × 100

(15) MBE=1n∑i=1n(yi − y^i)

(16) $$\mathrm{MAPE}(\%)=\frac{1}{n}\sum_{i=1}^{n}\left|\frac{y_{i}^{\prime }\;-\;y_{i}}{y_{i}}\right|\;\times \;100$$

(17) RMSE=1n∑i=1n(yi − y^i)2

where y_i_ is the actual value; y^i is the predicted value; ${\overset{¯}{y}}_{i}$ is the mean value of the dependent variable; and N is the number of samples in the test set.

### 2.7. Statistical Analysis

All experimental determinations were conducted with at least three biological replicates for each sample, and experimental data were expressed as mean ± standard deviation (SD). Origin 2021 was used for plotting, and MATLAB R2022b software was employed to model the artificial neural network. In total, approximately 60 channel catfish with an average weight of 2.00 kg were used in this study, yielding around 300 sample groups (each group consisting of three biological replicates).

## 3. Results and Discussion

### 3.1. Quality Analysis During Storage at Different Temperatures

**Figure 1:**
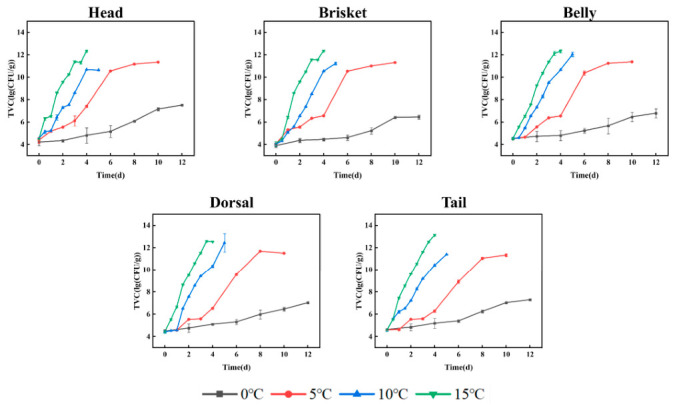
Change in TVC at different temperatures.

Changes in microbial populations are correlated with the degree of deterioration of aquatic products. As shown in Figure 1, the initial TVC in various regions of channel catfish was 3.75–4.61 log (CFU/g), which closely aligned with the values observed in the southern bluefin tuna [27] and rainbow trout [28]. These initial differences among muscle segments may be attributed to variations in anatomical location, such as proximity to visceral organs, which can influence the initial microbial load and contamination distribution. The total viable count exhibited an increase over the duration of storage, following an S-shaped trajectory. The rate of microbial proliferation exhibited variability across different environments. Microbial growth and reproduction accelerated with increasing temperature. According to the microbiological limit (≤7 log (CFU/g)) established for aquatic products by the International Commission on Microbiological Specifications for Foods [29], the HM and TM surpassed the permissible limit after 10 days of storage at 0 °C. Specifically, the BRM exhibited the lowest initial colony count of 3.89 log (CFU/g), and the TVC at 0 °C storage was markedly lower compared to that of the other segmentations of channel catfish. This may be associated with differences in tissue composition, such as moisture distribution, protein content, and microstructural characteristics, which can influence microbial growth environments [30]. The HM was preserved for 4 days at 5 °C, while the remaining segmentations exceeded the standard on the 6th day. The BRM exceeded the threshold at 2.5 days, while the rest of the segmentations surpassed the standard at 2 days under a temperature of 10 °C. The TM exceeded the standard at 1 day, with the other segmentations surpassing the standard at 1 day at 15 °C. These results further highlight segment-specific differences in spoilage dynamics, which are likely driven by variations in both physicochemical properties and microbial community distribution across different muscle regions. The findings demonstrated that storage at lower temperatures can effectively inhibit microbial activity [31].

**Figure 2:**
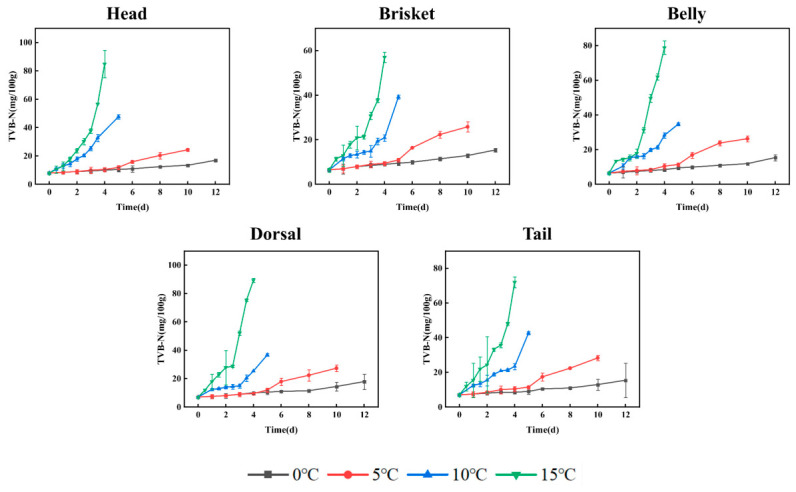
Change in TVB-N at different temperatures.

The enzymatic activity and bacterial action during refrigeration contribute to the degradation of proteins in aquatic products, leading to the formation of various volatile basic nitrogenous compounds [32]. Therefore, the measurement of TVB-N in channel catfish is widely regarded as an effective indicator of freshness. As shown in Figure 2, TVB-N levels gradually increased during the early stage of storage, followed by a significant surge in the later stages. Moreover, higher storage temperatures accelerated this increase. This phenomenon is likely due to enhanced microbial activity, intensive amino acid catabolism, and accelerated deamination processes [33]. According to hygienic standards, the acceptable limit for fish is set at 20 mg/100 g [2]. In this study, TVB-N values of all segmented parts of channel catfish remained below 20 mg/100 g after 12 days of storage at 0 °C. However, the values exceeded the international threshold on the 8th, 3rd, and 2nd day of storage at 5, 10, and 15 °C, respectively. These findings suggest that lower temperatures more effectively reduce endogenous enzymatic activity, inhibit microbial proliferation, and delay the accumulation of TVB-N, thereby slowing down protein degradation [34].

### 3.2. Predictive Models Based on TVC

#### 3.2.1. The First-Level Model

The modified Gompertz equation was employed to nonlinearly model the temporal variation in TVC across five segments of channel catfish stored at different temperature conditions. As illustrated in Table 1, the R^2^ for each fit exceeded 0.9, indicating that the model can accurately capture microbial growth dynamics across different catfish segments at various storage temperatures. Temperature exerted a substantial effect on lag phase duration (λ) and the maximum specific growth rate (μmax). As storage temperature increased, λ of microbial proliferation decreased, while both μ_max_ and the maximum microbial population (N_max_) exhibited an increase. This phenomenon was consistent with the values reported previously [35].

#### 3.2.2. Belehradek Modeling

**Figure 3:**
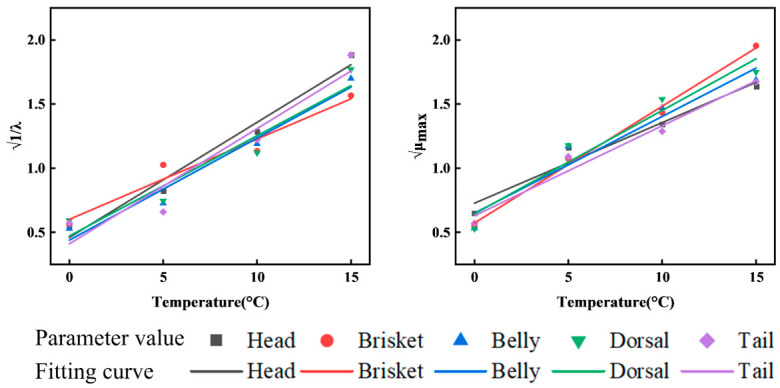
Relationship between temperature and microbial kinetic parameters.

Based on the primary model, the Belehradek equation was applied as the secondary model to describe the relationship between temperature and microbial growth kinetic parameters [36]. Within the temperature range of 0–15 °C, a strong linear correlation was observed between $\sqrt{\mu_{\max}}$ and $\sqrt{\frac{1}{\lambda }}$ (Figure 3). The application of Belehradek’s equation effectively captured the temperature dependence of microbial growth kinetics in various anatomical segments of channel catfish. The specific equations for the correlation between temperature, μ_max_, and λ were presented in Equations (18)–(22).

(18) $$\mathrm{Head}\;\sqrt{\mu_{\max}}=0.063(T-0.727)\;\sqrt{\frac{1}{\lambda }}=0.090(T-0.460)$$

(19) $$\mathrm{Brisket}\;\sqrt{\mu_{\max}}=0.091(T-0.572)\;\sqrt{\frac{1}{\lambda }}=0.063(T-0.601)$$

(20) $$\mathrm{Belly}\;\sqrt{\mu_{\max}}=0.075(T-0.649)\;\sqrt{\frac{1}{\lambda }}=0.080(T-0.439)$$

(21) $$\mathrm{Dorsal}\;\sqrt{\mu_{\max}}=0.081(T-0.644)\;\sqrt{\frac{1}{\lambda }}=0.078(T-0.471)$$

(22) $$\mathrm{Tail}\;\sqrt{\mu_{\max}}=0.070(T-0.629)\;\sqrt{\frac{1}{\lambda }}=0.090(T-0.410)$$

#### 3.2.3. Establishment and Evaluation of Microbiological Secondary Models

The maximum measured *N_max_* value of each anatomical segment at 15 °C (the upper limit of temperature fluctuation in actual cold-chain transportation) was adopted, and Equations (18)–(22) were incorporated into Equation (6) to establish a kinetic model for microbial growth at different temperatures. The final fitted equations are presented as Equations (23)–(27).

(23) $$\mathrm{Head}\;N\left(t_{i}\right)=N\left(t_{i-1}\right)+(12.71\;-\;N\left(t_{i-1}\right))\;\times \;\exp\{-\exp\left[\frac{0.063^{2}{\left(T\;-\;0.727\right)}^{2}\;\times \;2.718}{(12.71\;-\;N\left(t_{i-1}\right))}\;\times \;\left(\frac{1}{0.090^{2}{\left(T\;-\;0.460\right)}^{2}}\;-\;t_{i}\right)+1\right]\}$$

(24) $$\mathrm{Brisket}\;N(t_{i})=N(t_{i-1})+(12.94\;-\;N\left(t_{i-1}\right))\;\times \;\exp\{-\exp\left[\frac{0.091^{2}{(T\;-\;0.572)}^{2}\;\times \;2.718}{(12.94\;-\;N\left(t_{i-1}\right))}\;\times \;\left(\frac{1}{0.063^{2}{\left(T\;-\;0.601\right)}^{2}}\;-\;t_{i}\right)+1\right]\}$$

(25) $$\mathrm{Belly}\;N(t_{i})=N(t_{i-1})+(13.41\;-\;N\left(t_{i-1}\right))\;\times \;\exp\{-\exp\left[\frac{0.075^{2}{\left(T\;-\;0.649\right)}^{2}\;\times \;2.718}{(13.41\;-\;N\left(t_{i-1}\right))}\;\times \;\left(\frac{1}{0.080^{2}{\left(T\;-\;0.439\right)}^{2}}\;-\;t_{i}\right)+1\right]\}$$

(26) $$\mathrm{Dorsal}\;N(t_{i})=N(t_{i-1})+(13.32\;-\;N\left(t_{i-1}\right))\;\times \;\exp\{-\exp\left[\frac{0.081^{2}{\left(T\;-\;0.644\right)}^{2}\;\times \;2.718}{(13.32\;-\;N\left(t_{i-1}\right))}\;\times \;\left(\frac{1}{0.078^{2}{\left(T\;-\;0.471\right)}^{2}}\;-\;t_{i}\right)+1\right]\}$$

(27) $$\mathrm{Tail}\;N(t_{i})=N(t_{i-1})+(14.05\;-\;N\left(t_{i-1}\right))\;\times \;\exp\{-\exp\left[\frac{0.070^{2}{\left(T\;-\;0.629\right)}^{2}\;\times \;2.718}{(14.05\;-\;N\left(t_{i-1}\right))}\;\times \;\left(\frac{1}{0.090^{2}{\left(T\;-\;0.410\right)}^{2}}\;-\;t_{i}\right)+1\right]\}$$

When the channel catfish was stored at a constant temperature at 4 °C, the predicted TVC values for various sections of the channel catfish were calculated using Equations (23)–(27). A comparison of the predicted values against the measured values is shown in Table 2. The predictions demonstrated a high degree of accuracy within the initial 2 days of storage, exhibiting relative errors confined to ±15%. However, during the later stages of storage, the model exhibited systematic underprediction, with relative errors progressively increasing and, in some cases, exceeding 50%. This pronounced deviation may be attributed to the inherent limitations of the model. The spoilage of channel catfish is a complex and dynamic process, and the model may not fully capture the nonlinear interactions among microbial growth, substrate availability, and environmental factors [37]. In particular, as storage progresses, shifts in microbial communities, accumulation of metabolites, and potential secondary growth phases may accelerate microbial proliferation beyond the assumptions of the model. Additionally, the modified Gompertz equation, as a sigmoidal model, may inadequately describe the late-stage growth behavior or deviations from ideal growth patterns, leading to biased parameter estimation and reduced predictive reliability.

In practical applications, it was effective to monitor temperature variations, and the alterations of the TVC across various regions of channel catfish following temperature fluctuations can be obtained using Equations (23)–(27). To assess the model’s reliability, various sections of channel catfish were stored at fluctuating temperatures. The TVC was calculated and collected at specified time points (Table S1). The selected sampling points were intended to represent key stages during temperature fluctuation, capturing both intermediate and later responses of microbial growth to dynamic conditions. The REs for the different segments remained within ±15% (except for HM b). These models were considered satisfactory given the complexity of spoilage responses and the inherent variability [38]. The BRM exhibited the lowest REs within ±10%, highlighting the model’s effectiveness in forecasting TVC under the tested conditions. However, it should be noted that the validation was based on a limited number of sampling points under a specific fluctuation profile, which may not fully represent the continuous and diverse temperature variations encountered in real cold-chain scenarios. Therefore, further validation under more comprehensive and continuous dynamic temperature conditions is needed in future studies.

#### 3.2.4. Development and Evaluation of Microbiologically Based ANN Prediction Models

Training models for BPNN and RBFNN were developed using MATLAB’s newff and newrbe functions, respectively. The TVC data collected at 0, 5, 10, and 15 °C served as the training dataset for both models. As shown in Table 3, the R^2^ values for different segments exceeded 0.95, indicating an excellent fit. The MBE was maintained within ± 0.05, the MAPE was below 0.07, and the RMSE was under 0.6. These metrics suggested that the ANN models are capable of accurately predicting TVC in various parts of the channel catfish.

The TVC data collected at 0, 2, 4, 6, 8, and 10 days under 4 °C storage conditions served as the validation dataset, and the relative errors were assessed in comparison to the predicted values generated by the BFNN and RBFNN models. The performance of the ANN predictive model was systematically evaluated, with the findings presented in Table 4. For BPNN, the relative errors remained within ±10%, with the exception of the 2 days of HM (−13.82%). For the RBFNN model, the relative errors were maintained within ±20%, with the exception of TM 0–6 days and DM 2 days.

#### 3.2.5. Comparison of Residuals of Different Prediction Models for Microorganisms

**Figure 4:**
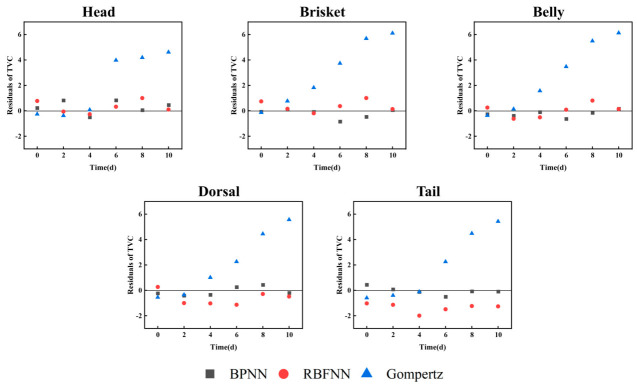
Residual difference between predicted and measured values of fish stored at 4°C for TVC.

Residual analyses of the modified Gompertz equations, the BPNN models, and the RBFNN models were performed using the TVC data obtained under storage conditions of 4 °C. As shown in Figure 4, the residuals of both the BPNN and RBFNN models exhibited a random and non-systematic distribution centered around zero, indicating that each data sample was independent. The absolute value of the residuals for the BPNN models across various segmentations was below 0.85, while the absolute value of the residuals for the RBFNN models did not exceed 2. The residuals of the modified Gompertz models exhibited an upward trend over time, with peak residuals reaching 6.12. Specifically, the predictive model in HM demonstrated compatibility with both the BPNN and RBFNN models, whereas the predictive model in BE exhibited optimal performance with the RBFNN model, yielding the lowest average residual (0.02). For other segments, the BPNN model generally outperformed the RBFNN model. Overall, the ANN-based models demonstrated superior predictive performance compared to the modified Gompertz equation. ANN-based approaches, including BPNN and RBFNN, are highly flexible and capable of capturing complex nonlinear relationships in microbial growth data, which explains their lower residuals and stronger predictive performance [39]. Nevertheless, these models are essentially data-driven black-box systems, and their internal parameters lack direct biological meaning, which limits mechanistic interpretation and reduces transparency in practical decision-making contexts.

By contrast, the kinetic modeling framework built on the modified Gompertz equation provides parameters with explicit biological significance, including lag phase duration (λ), maximum specific growth rate (μmax), and maximum population level (Nmax). These parameters can be directly linked to microbial adaptation and proliferation behavior under different environmental conditions. Furthermore, the incorporation of the Belehradek equation as a secondary model enables quantitative description of the temperature dependence of growth kinetics, allowing cross-temperature comparison and parameter-level interpretation. This structure enhances model transparency and supports mechanism-oriented analysis, shelf-life estimation, and risk assessment. Therefore, although the kinetic models showed slightly lower predictive accuracy than ANN-based models in the residual analysis, they offer substantial advantages in interpretability, parameter comparability, and theoretical relevance. Considering these complementary strengths, future work could explore hybrid or interpretable machine-learning strategies that integrate kinetic parameters with data-driven learning to improve predictive performance while maintaining model transparency.

### 3.3. Establishment of TVB-N Based Prediction Model

#### 3.3.1. Establishment of the Joint Arrhenius Equation for Primary Chemical Reaction Kinetics

A first-order kinetic equation was employed to nonlinearly analyze the temporal variation in TVB-N across five segments of channel catfish, maintained at different temperatures. The parameters were shown in Table S2, illustrating that the growth rate of TVB-N exhibited an exponential increase in relation to storage duration. The rate of increase varied across different temperature conditions, with higher temperatures leading to a faster accumulation of TVB-N. The model effectively characterized the dynamics of the increase in TVB-N across various regions of channel catfish subjected to different storage temperatures (R^2^ > 0.9). With the increase in storage temperature, the rate constant (K) of TVB-N generation also increased, suggesting that elevated temperatures promote protein degradation in channel catfish muscle. To further investigate the temperature dependence of the reaction, the Arrhenius equation was fitted to the rate constants obtained under different storage conditions. The resulting parameters, presented in Table S3, demonstrated a strong correlation (R^2^ > 0.95), indicating that the Arrhenius model provided a good fit for the temperature-dependent kinetic behavior.

#### 3.3.2. Secondary Modeling and Evaluation of Volatile Saline Nitrogen

The secondary model presented in Table S3 was incorporated into Equation (7) to obtain the growth kinetics models at fluctuating temperatures, as in (28)–(32)

(28) $$\mathrm{Head}\;B\left(t_{i}\right)=B_{i-1}e^{1.10\times 1015\exp(-\frac{84254.97}{\mathrm{RT}})t_{i}}$$

(29) $$\mathrm{Brisket}\;B\left(t_{i}\right)=B_{i-1}e^{4.72\times 1013\exp(-\frac{76878.37}{\mathrm{RT}})t_{i}}$$

(30) $$\mathrm{Belly}\;B\left(t_{i}\right)={\;B}_{i-1}e^{1.19\times 1015\exp(-\frac{84227.73}{\mathrm{RT}})t_{i}}$$

(31) $$\mathrm{Dorsal}\;B\left(t_{i}\right)=B_{i-1}e^{4.44\times 1015\exp(-\frac{87361.99}{\mathrm{RT}})t_{i}}$$

(32) $$\mathrm{Tail}\;B\left(t_{i}\right)={\;B}_{i-1}e^{2.96\times 1014\exp(-\frac{81094.80}{\mathrm{RT}})t_{i}}$$

In practice, channel catfish samples were stored at 4 °C, enabling the prediction of TVB-N levels for various anatomical segments using Equations (28)–(32). A comparative analysis was presented in Table 2. With the exception of the 0-day measurement, most relative errors for the different segments were within ±15%, with only three values slightly exceeding this range (−17.87, −17.48, and −16.28). Overall, the model demonstrated acceptable predictive performance for TVB-N levels across different channel catfish segments under varying temperature conditions.

The TVB-N was measured at designated intervals during the fluctuating temperature experiment, and the results were compared with the predicted results, as shown in Table S1. The relative errors were maintained within ±15% (with the exception of point b in HM and BEM), indicating strong model reliability under dynamic temperature conditions. In particular, the relative errors of the BRM, BEM, and TM at both sampling points were within ±5%, indicating that the model demonstrated better reliability in predicting the muscles in the above three segments. In conclusion, the developed models of TVB-N dynamics under fluctuating temperatures can effectively predict the changing state of the TVB-N across various regions of channel catfish during actual storage and transportation. In practice, channel catfish quality can be predicted in real time based on data on temperature conditions along the supply chain [40].

#### 3.3.3. The Early Warning Threshold and Evaluation of Shelf Life

Equation (8) represents the slope of a first-order kinetic curve: When the slope reaches a certain threshold, the curve exhibits a rapid increase, indicating a critical point that can be defined as the early warning threshold for the TVB-N model. By monitoring the rate of change in TVB-N content, it is possible to predict quality deterioration in a timely manner, thereby providing a scientific basis for the storage and preservation of aquatic products. By substituting the values from Table S2 into Equation (8), the instantaneous rate of change in TVB-N over time was obtained, reflecting the dynamic spoilage process of channel catfish during storage. This analysis enables the identification of the critical point at which the rate of change in TVB-N content reaches a specific threshold, thereby establishing an early warning threshold (Table S4). Specifically, the early warning thresholds for TVB-N content in the head, mesenteric, abdominal, dorsal, and caudal segments were determined to be 9.15, 8.40, 7.99, 8.04, and 8.47 mg/100 g, respectively. Due to differences in spoilage mechanisms and rates, early warning thresholds vary among different types of aquatic products. Even within the same species, different anatomical segments can exhibit distinct threshold values. Establishing an early warning time-point model for channel catfish is crucial to ensuring food safety and minimizing economic losses. By determining early spoilage thresholds, timely interventions can be implemented to prevent economic damage caused by the deterioration of segmented products during storage.

The shelf life of different anatomical segments of channel catfish was estimated based on the TVB-N first-order chemical reaction kinetic model over the temperature range of 0–15 °C [11]. Specifically, shelf life was calculated using Equation (1.10), which was derived by incorporating the temperature-dependent secondary model into the primary kinetic model. The recorded shelf-life values at 0–15 °C were utilized to assess the precision of this kinetic prediction model. Based on hygienic standards, a TVB-N concentration of 20 mg/100 g is generally considered the acceptable limit for fish [2]. The shelf life of channel catfish was calculated using Equations (33)–(37) and subsequently compared with the measured values, with the findings presented in Table 5. The relative error across various segments at different temperatures remained within ±20% (except for HM at 0 °C storage), suggesting that the predictive model demonstrated a high level of accuracy. Furthermore, a decrease in storage temperature correlated with an extended shelf life.

(33) $$\mathrm{Head}\;\mathrm{SL}=\frac{\ln(\frac{B}{B_{0}})}{1.10\;\times \;10^{15}\exp(-\frac{84254.97}{\mathrm{RT}})}$$

(34) $$\mathrm{Brisket}\;\mathrm{SL}=\frac{\ln(\frac{B}{B_{0}})}{4.72\;\times \;10^{13}\exp(-\frac{76878.37}{\mathrm{RT}})}$$

(35) $$\mathrm{Belly}\;\mathrm{SL}=\frac{\ln(\frac{B}{B_{0}})}{1.19\;\times {\;10}^{15}\exp(-\frac{84227.73}{\mathrm{RT}})}$$

(36) $$\mathrm{Dorsal}\;\mathrm{SL}=\frac{\ln(\frac{B}{B_{0}})}{4.44\;\times \;10^{15}\exp(-\frac{87361.99}{\mathrm{RT}})}$$

(37) $$\mathrm{Tail}\;\mathrm{SL}=\frac{\ln(\frac{B}{B_{0}})}{2.96\;\times \;10^{14}\exp(-\frac{81094.80}{\mathrm{RT}})}$$

#### 3.3.4. Development and Evaluation of the ANN Prediction Models

The TVB-N data obtained at the temperature of 0–15 °C were employed to train the BPNN and RBFNN models. The outcomes of this training are illustrated in the accompanying Table 6. Both models demonstrated a satisfactory fitting degree (R^2^ > 0.8). Additionally, the MBE of the BPNN was maintained within ±0.9, the MAPE was less than 0.5, and the RMSE was below 6.5. These results indicate that the BPNN and RBFNN models were capable of accurately predicting TVB-N of different parts of channel catfish.

The BPNN and RVNN predictive models were established using TVB-N data collected at different temperature conditions. The TVB-N values recorded at 0, 2, 4, 6, 8, and 10 days under 4 °C storage conditions served as test datasets, and the REs were analyzed with the predicted values of the ANN predictive model. As shown in Table 7, the REs predominantly fell within ±15%. It indicated that the BPNN models were suitable for the prediction of various segments of channel catfish. However, during the early storage period (0–2 days), substantially larger deviations were observed in RBFNN models, with the maximum error exceeding 80%. This large deviation may reflect an inherent limitation of the RBFNN model, which is related to its structural characteristics and sensitivity to data distribution. At the initial stage of storage, the number of data points is limited and the variation range is relatively narrow, which may lead to insufficient distribution of radial basis functions in this region. In addition, RBFNN is more sensitive to measurement fluctuations or noise at low concentration levels, which may further amplify prediction errors [39].

#### 3.3.5. Comparison of Residuals of Different Prediction Models for TVB-N

**Figure 5:**
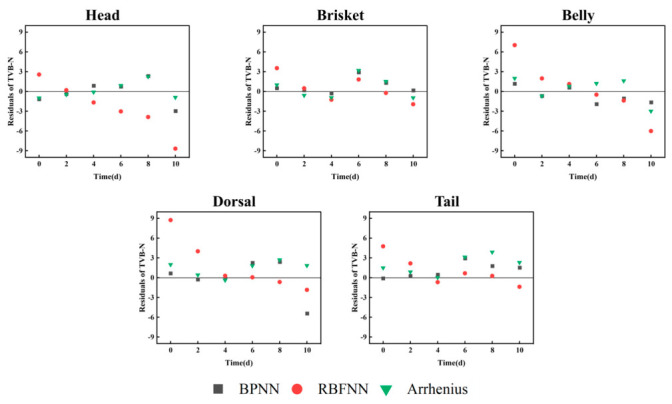
Residual difference between predicted and measured values of fish stored at 4°C for TVB-N.

Residual analyses were performed for the Arrhenius equation, the BPNN model, and the RBFNN model using TVB-N data under 4 °C storage conditions. As shown in Figure 5, the residuals from all three models were randomly and symmetrically distributed around zero, indicating that the data points were independent and that the models did not exhibit systematic bias. The absolute residuals for the BPNN model across various segments were all below 5.5, while those for the RBFNN model were under 8.9, and for the Arrhenius equation, less than 3.9. Among the three models, the Arrhenius equation showed the best overall residual performance. Notably, this does not necessarily indicate that it is the most suitable model for predicting quality changes in segments of channel catfish, as different models exhibit distinct applicability across scenarios. For example, the HM and DM segments were well predicted by both the BPNN model and the Arrhenius equation, whereas the remaining segments were more compatible with the BPNN model. Artificial neural network models, particularly the BPNN model, have demonstrated strong adaptability across various fields. This endows them with robust capability in capturing the nonlinear and segment-dependent variations in the evolution of TVB-N. Consequently, they hold significant potential for more accurate and rapid determination of critical early-warning thresholds for different aquatic products in future studies. In contrast, although the Arrhenius equation is structurally simple, it offers clear physicochemical interpretability by explicitly linking deterioration rates with temperature. This mechanistic basis enables parameter-level insight and allows extrapolation within a defined temperature range. In practice, such temperature–rate relationships are valuable for quality risk warning and threshold-based decision-making, as they directly reflect the impact of temperature fluctuations on spoilage progression. In future work, the complementary application of these two approaches is expected to further enhance the reliability of early warning.

## 4. Conclusions

In this study, two freshness indicators (TVB-N and TVC) were evaluated across various anatomical segments of channel catfish stored under different temperature conditions. A predictive and early-warning model was developed to monitor quality dynamics throughout storage. The TVB-N and TVC levels in different segments exhibited distinct rates of change with increasing storage time and temperature, reflecting segment-specific kinetic characteristics. The established models followed first-order kinetic behavior, and among the tested approaches, the BPNN model demonstrated superior predictive performance, with relative errors generally within ±15%. Under various temperature conditions, the relative errors between measured and predicted values also remained within ±15%. Within the validation range of 0–15 °C, the predictive models accurately estimated the storage duration, with prediction errors within ±20%. Moreover, early warning thresholds for sharp quality changes in individual segments were determined from the rate of change in TVB-N content, providing valuable technical guidance for process control.

Overall, the developed models contribute to a deeper understanding of temperature-dependent quality dynamics and offer a scientific basis for optimizing quality regulation and intelligent cold chain management. It should be noted that each model has inherent limitations—ANN models offer high predictive accuracy but rely heavily on available data, while kinetic models provide mechanistic insight but may not capture complex interactions or environmental variability. In future studies, we will further attempt to establish a link between microbial community structure and kinetic modeling, aiming to enhance both the mechanistic understanding and predictive capability of spoilage models and improve model applicability under diverse storage conditions.

## Figures and Tables

**Table 1:** Microbial growth parameters.

Temperature (°C)	Segmentation	N_max_/(1og(CFU/g))	μ_max_/d^–1^	λ/d	R^2^
0	Head	9.04	0.42	3.43	0.985
	Brisket	9.64	0.31	3.21	0.917
	Belly	8.54	0.28	3.59	0.985
	Dorsal	9.77	0.28	2.84	0.991
	Tail	8.69	0.32	3.05	0.978
5	Head	11.86	1.35	1.47	0.974
	Brisket	12.36	1.15	0.95	0.939
	Belly	12.02	1.37	1.90	0.965
	Dorsal	12.45	1.39	1.80	0.975
	Tail	12.45	1.19	2.31	0.982
10	Head	11.91	1.80	0.61	0.972
	Brisket	12.94	2.05	0.78	0.995
	Belly	12.95	2.16	0.71	0.993
	Dorsal	12.80	2.37	0.80	0.969
	Tail	13.17	1.66	0.67	0.988
15	Head	12.71	2.68	0.28	0.974
	Brisket	12.24	3.82	0.41	0.990
	Belly	13.41	2.85	0.35	0.996
	Dorsal	13.32	3.06	0.32	0.992
	Tail	14.05	2.80	0.28	0.987

**Table 2:** Predicted and measured values of catfish stored at 4 °C.

Index	Segmentation	Time (d)	0	2	4	6	8	10
TVC	Head	Predictive value	6.33	6.36	6.39	6.43	6.47	6.51
		Measured value	6.07	5.98	6.47	10.41	10.65	11.12
		RE (%)	4.35	6.41	−1.11	−38.23	−39.30	−41.48
	Brisket	Predictive value	4.65	4.66	4.68	4.71	4.74	4.77
		Measured value	4.51	5.43	6.50	8.44	10.42	10.88
		RE (%)	3.07	−14.13	−28.00	−44.23	−54.56	−56.14
	Belly	Predictive value	4.79	4.83	4.87	4.92	4.97	5.03
		Measured value	4.42	4.96	6.44	8.39	10.47	11.16
		RE (%)	8.35	−2.68	−24.34	−41.35	−52.50	−54.93
	Dorsal	Predictive value	4.77	4.81	4.85	4.90	4.96	5.02
		Measured value	4.22	4.45	5.86	7.16	9.40	10.58
		RE (%)	13.03	7.96	−17.25	−31.53	−47.27	−52.60
	Tail	Predictive value	4.84	4.90	4.95	5.01	5.08	5.14
		Measured value	4.24	4.50	4.84	7.26	9.56	10.57
		RE (%)	14.05	8.87	2.42	−30.98	−46.89	−51.34
TVB-N	Head	Predictive value	6.94	7.91	10.52	13.99	18.59	24.72
		Measured value	5.95	7.44	10.42	14.88	20.83	23.81
		RE (%)	16.67	6.35	0.99	−6.02	−10.76	3.80
	Brisket	Predictive value	5.95	8.04	10.86	14.67	19.81	26.76
		Measured value	6.94	7.44	9.92	17.86	21.33	25.80
		RE (%)	−14.29	8.05	9.46	−17.87	−7.12	3.73
	Belly	Predictive value	5.95	8.13	11.10	15.16	20.71	28.28
		Measured value	7.94	7.44	11.91	16.37	22.32	25.30
		RE (%)	−25.00	9.26	−6.74	−7.37	−7.23	11.80
	Dorsal	Predictive value	5.95	8.02	10.81	14.56	19.62	26.44
		Measured value	7.94	8.43	10.42	16.37	22.32	28.28
		RE (%)	−25.00	−4.89	3.75	−11.04	−12.09	−6.49
	Tail	Predictive value	5.95	8.05	10.89	14.74	19.94	26.97
		Measured value	7.44	8.93	10.91	17.86	23.81	29.27
		RE (%)	−20.00	−9.81	−0.18	−17.48	−16.28	−7.86

**Table 3:** The training set predicts the effect of TVC.

ANN	Segmentation	R^2^	MBE	MAPE	RMSE
BPNN	Head	0.987	−0.040	0.034	0.294
	Brisket	0.983	0.024	0.048	0.363
	Belly	0.987	−0.045	0.035	0.304
	Dorsal	0.987	0.036	0.037	0.327
	Tail	0.991	−0.049	0.023	0.244
RBFNN	Head	0.987	0.000	0.031	0.295
	Brisket	0.958	−0.009	0.062	0.569
	Belly	0.971	−0.005	0.049	0.454
	Dorsal	0.966	−0.007	0.055	0.515
	Tail	0.995	0.001	0.023	0.182

**Table 4:** Predicted and measured values of the TVC for catfish stored at 4 °C.

ANN	Segmentation	Time (d)	0	2	4	6	8	10
BPNN	Head	Predictive value	5.84	5.15	6.96	9.57	10.59	10.65
		Measured value	6.07	5.98	6.47	10.41	10.65	11.12
		RE (%)	−3.80	−13.82	7.69	−8.01	−0.58	−4.18
	Brisket	Predictive value	4.58	5.32	6.58	9.28	10.89	10.81
		Measured value	4.51	5.43	6.50	8.44	10.42	10.88
		RE (%)	1.43	−2.00	1.16	9.95	4.47	−0.61
	Belly	Predictive value	4.68	5.35	6.54	9.02	10.62	10.98
		Measured value	4.42	4.96	6.44	8.39	10.47	11.16
		RE (%)	5.76	7.72	1.47	7.48	1.47	−1.53
	Dorsal	Predictive value	4.44	4.85	6.21	6.89	8.96	10.77
		Measured value	4.22	4.45	5.86	7.16	9.40	10.58
		RE (%)	5.32	9.01	5.85	−3.70	−4.68	1.82
	Tail	Predictive value	3.80	4.41	4.94	7.76	9.63	10.65
		Measured value	4.24	4.50	4.84	7.26	9.56	10.57
		RE (%)	−10.57	−1.87	2.23	6.78	0.74	0.83
RBFNN	Head	Predictive value	5.29	6.04	6.73	10.09	9.66	11.02
		Measured value	6.07	5.98	6.47	10.41	10.65	11.12
		RE (%)	−12.75	1.02	4.15	−3.08	−9.38	−0.92
	Brisket	Predictive value	3.77	5.28	6.70	8.07	9.42	10.75
		Measured value	4.51	5.43	6.50	8.44	10.42	10.88
		RE (%)	−16.47	−2.85	3.01	−4.35	−9.60	−1.23
	Belly	Predictive value	4.17	5.59	6.96	8.31	9.66	11.02
		Measured value	4.42	4.96	6.44	8.39	10.47	11.16
		RE (%)	−5.69	12.67	8.01	−1.01	−7.68	−1.20
	Dorsal	Predictive value	3.95	5.46	6.89	8.29	9.68	11.07
		Measured value	4.22	4.45	5.86	7.16	9.40	10.58
		RE (%)	−6.30	22.52	17.50	15.84	3.04	4.57
	Tail	Predictive value	5.27	5.63	6.83	8.75	10.79	11.83
		Measured value	4.24	4.50	4.84	7.26	9.56	10.57
		RE (%)	24.17	25.32	41.19	20.49	12.92	11.93

**Table 5:** Predicted and measured values for the shelf life of catfish.

Temperature (°C)	Shelf Life (d)	Head	Brisket	Belly	Dorsal	Tail
0	Predictive value	11.10	12.28	12.41	12.37	11.74
	Measured value	14	14	14	14	14
	RE (%)	−26.17	−14.01	−12.82	−13.13	−19.29
4	Predictive value	8.82	9.28	8.63	8.71	8.90
	Measured value	8	9	10	8	10
	RE (%)	9.27	2.99	−15.86	8.12	−12.31
5	Predictive value	7.73	8.23	7.57	7.60	7.84
	Measured value	8	8	8	8	8
	RE (%)	−3.49	2.76	−5.72	−5.31	−1.98
10	Predictive value	2.99	3.71	3.34	3.18	3.32
	Measured value	3	4	3.5	3.5	3.5
	RE (%)	−0.37	−7.81	−4.66	−10.20	−5.40
15	Predictive value	1.68	2.08	1.76	1.63	1.80
	Measured value	2	2	2	1.5	2
	RE (%)	−18.81	3.95	−13.48	7.77	−11.38

**Table 6:** The training set predicts the effect for TVB-N.

ANN	Segmentation	R2	MBE	MAPE	RMSE
BPNN	Head	0.946	−0.385	0.113	3.403
	Brisket	0.886	−0.703	0.152	3.741
	Belly	0.921	−0.372	0.208	4.354
	Dorsal	0.975	0.120	0.118	2.842
	Tail	0.905	0.852	0.135	4.168
RBFNN	Head	0.808	0.000	0.306	6.426
	Brisket	0.848	0.000	0.218	4.148
	Belly	0.834	0.000	0.353	6.306
	Dorsal	0.830	0.000	0.389	7.436
	Tail	0.857	0.000	0.249	5.111

**Table 7:** Predicted and measured values of the TVB-N for catfish stored at 4 °C.

ANN	Segmentation	Time (d)	0	2	4	6	8	10
BPNN	Head	Predictive value	7.12	7.81	9.53	14.13	18.48	26.76
		Measured value	5.95	7.44	10.42	14.88	20.83	23.81
		RE (%)	19.53	4.95	−8.51	−5.03	−11.31	12.39
	Brisket	Predictive value	6.44	7.23	10.20	14.95	20.02	25.63
		Measured value	6.94	7.44	9.92	17.86	21.33	25.80
		RE (%)	−7.33	−2.80	2.78	−16.27	−6.16	−0.64
	Belly	Predictive value	6.76	8.11	11.31	18.27	23.38	26.93
		Measured value	7.94	7.44	11.91	16.37	22.32	25.30
		RE (%)	−14.79	8.95	−5.04	11.61	4.72	6.45
	Dorsal	Predictive value	7.27	8.70	10.27	14.12	19.92	33.69
		Measured value	7.94	8.43	10.42	16.37	22.32	28.28
		RE (%)	−8.35	3.11	−1.45	−13.77	−10.75	19.16
	Tail	Predictive value	7.54	8.66	10.45	14.92	22.03	27.74
		Measured value	7.44	8.93	10.91	17.86	23.81	29.27
		RE (%)	1.31	−3.04	−4.27	−16.48	−7.49	−5.24
RBFNN	Head	Predictive value	3.39	7.25	12.09	17.91	24.71	32.49
		Measured value	5.95	7.44	10.42	14.88	20.83	23.81
		RE (%)	−43.12	−2.60	16.05	20.35	18.61	36.46
	Brisket	Predictive value	3.41	6.97	11.18	16.04	21.56	27.73
		Measured value	6.94	7.44	9.92	17.86	21.33	25.80
		RE (%)	−50.91	−6.39	12.64	−10.18	1.07	7.51
	Belly	Predictive value	0.91	5.47	10.78	16.86	23.70	31.31
		Measured value	7.94	7.44	11.91	16.37	22.32	25.30
		RE (%)	−88.52	−26.54	−9.43	3.00	6.18	23.74
	Dorsal	Predictive value	0.80	4.43	10.13	16.32	22.98	30.12
		Measured value	7.94	8.43	10.42	16.37	22.32	28.28
		RE (%)	−89.92	−47.52	−2.75	−0.33	2.95	6.54
	Tail	Predictive value	2.68	6.76	11.60	17.19	23.54	30.65
		Measured value	7.44	8.93	10.91	17.86	23.81	29.27
		RE (%)	−63.93	−24.27	6.27	−3.74	−1.13	4.73

## Data Availability

The original contributions presented in this study are included in the article/supplementary material. Further inquiries can be directed to the corresponding authors.

## Supplementary Materials

The following supporting information can be downloaded at: https://www.mdpi.com/article/10.3390/foods15091557/s1. Table S1. Predicted and measured values under fluctuating temperatures in catfish; Table S2. Kinetic modelling parameters for the variation of TVB-N values in catfish under different storage temperature conditions; Table S3. Relation parameters between temperature and rate of change. Table S4. Early warning value of channel catfish under different temperature conditions. Figure S1. Schematic diagram of fine segmentation of channel catfish.

## References

[1] Lin H.M., Hung Y.C., Deng S.G. (2020). Effect of partial replacement of polyphosphate with alkaline electrolyzed water (AEW) on the quality of catfish fillets. Food Control.

[2] Huang H., Sun W., Xiong G., Shi L., Jiao C., Wu W., Li X., Qiao Y., Liao L., Ding A. (2020). Effects of HVEF treatment on microbial communities and physicochemical properties of catfish fillets during chilled storage. LWT—Food Sci. Technol..

[3] Li B., Liu S., Chen X., Su Y., Pan N., Liao D., Qiao K., Chen Y., Liu Z. (2023). Dynamic Changes in the Microbial Composition and Spoilage Characteristics of Refrigerated Large Yellow Croaker (*Larimichthys crocea*) during Storage. Foods.

[4] Duan X., Li Z., Wang L., Lin H., Wang K. (2022). Engineered nanomaterials-based sensing systems for assessing the freshness of meat and aquatic products: A state-of-the-art review. Compr. Rev. Food Sci. Food Saf..

[5] Huang J., Wang L., Zhu Z., Zhang Y., Xiong G., Li S. (2023). Three Phenolic Extracts Regulate the Physicochemical Properties and Microbial Community of Refrigerated Channel Catfish Fillets during Storage. Foods.

[6] Geng Z., Shang D., Han Y., Zhong Y. (2019). Early warning modeling and analysis based on a deep radial basis function neural network integrating an analytic hierarchy process: A case study for food safety. Food Control.

[7] Lan W., Yang X., Gong T., Xe J. (2023). Predicting the shelf life of *Trachinotus ovatus* during frozen storage using a back propagation (BP) neural network model. Aquac. Fish..

[8] Zhang L., Li X., Lu W., Shen H., Luo Y. (2011). Quality predictive models of grass carp (*Ctenopharyngodon idellus)* at different temperatures during storage. Food Control.

[9] Liu X., Jiang Y., Shen S., Luo Y., Gao L. (2015). Comparison of Arrhenius model and artificial neuronal network for the quality prediction of rainbow trout (*Oncorhynchus mykiss*) fillets during storage at different temperatures. LWT—Food Sci. Technol..

[10] Xu Z., Liu X., Wang H., Hong H., Luo Y. (2017). Comparison between the Arrhenius model and the radial basis function neural network (RBFNN) model for predicting quality changes of frozen shrimp (*Solenocera melantho*). Int. J. Food Prop..

[11] Genç I.Y., Diler A. (2019). Development of Shelf Life Prediction Model in Rainbow Trout Stored at Different Temperatures. J. Aquat. Food Prod. Technol..

[12] Hosseini S.V., Pero M., Hoseinabadi Z., Tahergorabi R., Kazemzadeh S., Alemán R.S., Fuentes J.A.M., Fernández I.M., Calderon D.P., Sanchez X.F. (2023). Sous-vide processing of silver carp: Effect of processing temperature and cold storage duration on the microbial quality of the product as well as modeling by artificial neural networks. PLoS ONE.

[13] Kong C., Duan C., Zhang Y., Shi C., Luo Y. (2023). Changes in Lipids and Proteins of Common Carp (*Cyprinus carpio*) Fillets under Frozen Storage and Establishment of a Radial Basis Function Neural Network (RBFNN). Foods.

[14] Niu Y., Ye L., Shi Y., Gu H., Luo A. (2025). Development of shelf-life prediction models and programs for ‘Xuxiang’ kiwifruit stored at different temperatures. Postharvest Biol. Technol..

[15] Malak M.N.L., Abdel-Naeem H.H.S., Abdelsalam A.A., Ezzat G.A. (2025). A comparative study concerning the sensory, physicochemical, bacteriological, nutritional quality, heavy metal content, and health risk assessment of some low-cost fish species. Food Control.

[16] Short E.I. (1954). The Estimation of Total Nitrogen Using the Conway Micro-diffusion Cell. J. Clin. Pathol..

[17] Huang L. (2011). A new mechanistic growth model for simultaneous determination of lag phase duration and exponential growth rate and a new Belehdradek-type model for evaluating the effect of temperature on growth rate. Food Microbiol..

[18] Zwietering M.H., Jongenburger I., Rombouts F.M., Riet T. (1990). Modeling of the bacterial growth curve. Appl. Environ. Microbiol..

[19] Ratkowsky D.A., Olley J., McMeekin T.A., Ball A. (1982). Relationship between temperature and growth rate of bacterial cultures. J. Bacteriol..

[20] Bruckner S., Albrecht A., Petersen B., Kreyenschmidt J. (2013). A predictive shelf life model as a tool for the improvement of quality management in pork and poultry chains. Food Control.

[21] Mai T.N., Gudjónsdottir M., Lauzon H.L., Sveinsdóttir K., Martinsdóttir E., Audorff H., Reichstein W., Haarer D., Bogason S.G., Arason S. (2011). Continuous quality and shelf life monitoring of retail-packed fresh cod loins in comparison with conventional methods. Food Control.

[22] McMeekin T., Bowman J., McQuestin O., Mellefont L., Ross T., Tamplin M. (2008). The future of predictive microbiology: Strategic research, innovative applications and great expectations. Int. J. Food Microbiol..

[23] Wang H., Zheng Y., Shi W., Wang X. (2022). Comparison of Arrhenius model and artificial neuronal network for predicting quality changes of frozen tilapia (*Oreochromis niloticus*). Food Chem..

[24] Taoukis P.S., Koutsoumanis K., Nychas G.J.E. (1999). Use of time temperature integrators and predictive modelling for shelf life control of chilled fish under dynamic storage conditions. Int. J. Food Microbiol..

[25] Shao X., Guo Z., Qin Y., Zhao J., Guo Y., Sun X., Du F. (2025). Synergistic multi-level fusion framework of VNIR and SWIR hyperspectral data for soybean fungal contamination detection. Food Chem..

[26] Huang X., Wang H., Qu S., Luo W., Gao Z. (2021). Using artificial neural network in predicting the key fruit quality of loquat. Food Sci. Nutr..

[27] Bu Y., Han M., Tan G., Zhu W., Li X., Li J. (2022). Changes in quality characteristics of southern bluefin tuna (*Thunnus maccoyii*) during refrigerated storage and their correlation with color stability. LWT—Food Sci. Technol..

[28] Li Q., Lv J., Zhang L., Dong Z., Feng L., Luo Y. (2017). Biogenic Amines and Predictive Models of Quality of Rainbow Trout (*Oncorhynchus mykiss*) Fillets during Storage. J. Food Prot..

[29] International Commission on Microbiological Specifications for Foods—ICMSF (1986). Microorganisms in Foods 2: Sampling for Microbiological Analysis: Principles and Specific Applications.

[30] Li W., Jiang H., Wang B., Gong H., Lin X., Ji C., Zhang S. (2025). Quality and microbiome changes in different muscles of channel catfish (*Ictalurus punctatus*) during cold storage. Food Compos. Anal..

[31] Zhuang S., Hong H., Zhang L., Luo Y. (2021). Spoilage-related microbiota in fish and crustaceans during storage: Research progress and future trends. Compr. Rev. Food Sci. Food Saf..

[32] Tavares J., Martins A., Fidalgo L.G., Lima V., Amaral R.A., Pinto C.A., Silva A.M., Saraiva J.A. (2021). Fresh fish degradation and advances in preservation using physical emerging technologies. Foods.

[33] Liu Z., Liu Q., Wei S., Sun Q., Xia Q., Zhang D., Shi W., Ji H., Liu S. (2021). Quality and volatile compound analysis of shrimp heads during different temperature storage. Food Chem. X.

[34] Bekhit A.E.D.A., Holman B.W.B., Giteru S.G., Hopkins D.L. (2021). Total volatile basic nitrogen (TVB-N) and its role in meat spoilage: A review. Trends Food Sci. Technol..

[35] Park S.Y., Choi S.Y., Ha S.D. (2019). Predictive Modeling for the Growth of *Aeromonas hydrophila* on Lettuce as a Function of Combined Storage Temperature and Relative Humidity. Foodborne Pathog. Dis..

[36] Li Z., Wen Y., Yan Y., Ning Y., Xie M., Zhu Y., Wang H. (2024). The kinetic study on key quality and microbial content of fresh-cutting Chinese yams (*Dioscorea opposita* Thunb.) at different storage temperatures. J. Stored Prod. Res..

[37] Odeyemi O.A., Alegbeleye O.O., Strateva M., Stratev D. (2020). Understanding spoilage microbial community and spoilage mechanisms in foods of animal origin. Compr. Rev. Food Sci. Food Saf..

[38] Zhuang S., Liu Y., Gao S., Tan Y., Hong H., Luo Y. (2023). Mechanisms of fish protein degradation caused by grass carp spoilage bacteria: A bottom-up exploration from the molecular level, muscle microstructure level, to related quality changes. Food Chem..

[39] Shi X., Zhang J., Shi C., Tan Y., Hong H., Luo Y. (2022). Nondestructive prediction of freshness for bighead carp (*Hypophthalmichthys nobilis*) head by Excitation-Emission Matrix (EEM) analysis based on fish eye fluid: Comparison of BPNNs and RBFNNs. Food Chem..

[40] Corradini M.G. (2018). Shelf Life of Food Products: From Open Labeling to Real-Time Measurements. Annu. Rev. Food Sci. Technol..

