# Supplementary material for: Predictive Modeling of Channel Catfish Under Varying Temperatures: Quality Dynamics and Warning Thresholds
Source: Foods. 2026 Apr 30;15(9):1557. doi: 10.3390/foods15091557 (PMC13163288; doi:10.3390/foods15091557)
Supplement: Supplementary file 1 [file foods-15-01557-s001.zip › foods-4161716-supplementary.pdf]

Supplementary Material

Table S1. Predicted and measured values under fluctuating temperatures in catfish

Sampling point	Segmentation	Head	Brisket	Belly	Dorsal	Tail
	Predictive value	8.07	8.49	8.22	8.17	8.01
a-TVC	Measured value	9.31	9.35	9.43	9.30	9.24
	RE (%)	-13.34	-9.19	-12.86	-12.15	-13.28
	Predictive value	9.63	11.14	10.22	10.38	9.99
b-TVC	Measured value	11.70	11.44	11.26	11.34	11.57
	RE (%)	-17.66	-2.64	-9.24	-8.47	-13.64
	Predictive value	22.85	13.38	16.11	16.91	20.17
a-TVB-N	Measured value	24.80	13.89	14.39	16.37	20.34
	RE (%)	-7.87	-3.66	11.98	3.27	-0.82
	Predictive value	93.15	53.28	74.97	77.09	85.66
b-TVB-N	Measured value	135.43	52.58	93.26	78.88	86.81
	RE (%)	-31.22	1.32	-19.61	-2.26	-1.32

Table S2 Kinetic modelling parameters for the variation of TVB-N values in catfish under different storage temperature conditions

Temperature (°C)	Segmentation	Head	Brisket	Belly	Dorsal	Tail
0	K	0.058	0.072	0.069	0.075	0.063
	R^2	0.974	0.990	0.977	0.973	0.978
5	K	0.111	0.142	0.146	0.138	0.140
	R^2	0.990	0.990	0.980	0.985	0.990
10	K	0.351	0.340	0.350	0.324	0.351
	R^2	0.987	0.918	0.928	0.954	0.935
15	K	0.574	0.532	0.636	0.652	0.577
	R^2	0.980	0.968	0.979	0.973	0.970

Table S3 Relation parameters between temperature and rate of change

Segmentation	K_0（×10^{15}）	Ea	Formula	R^2
Head	1.10	84254.97	$K_B=1.10\times10^{15}\exp(-\frac{84254.97}{RT})$	0.955
Brisket	0.0472	76878.37	$K_B=4.72\times10^{13}\exp(-\frac{76878.37}{RT})$	0.972
Belly	1.19	84227.73	$K_B=1.19\times10^{15}\exp(-\frac{84227.73}{RT})$	0.987
Dorsal	4.44	87361.99	$K_B=4.44\times10^{15}\exp(-\frac{87361.99}{RT})$	0.988
Tail	0.296	81094.80	$K_B=2.96\times10^{14}\exp(-\frac{81094.80}{RT})$	0.974

Table S4 Early warning value of channel catfish under different temperature conditions

Segmentation	0°C	5°C	10°C	15°C	Threshold (mg·100g^{-1})
Head	10.34	9.01	8.55	8.71	9.15
Brisket	8.33	7.04	8.82	9.40	8.40
Belly	8.70	6.85	8.57	7.86	7.99
Dorsal	8.00	7.25	9.26	7.67	8.04
Tail	9.52	7.14	8.55	8.67	8.47

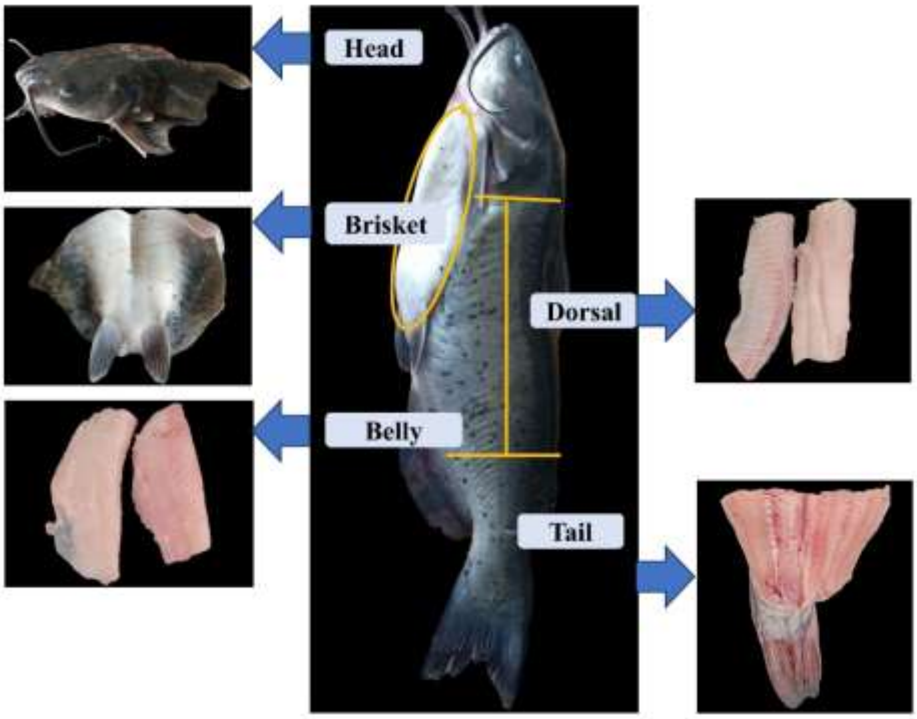

Figure S1. Schematic diagram of fine segmentation of channel catfish
